# Application of CMT-Twin DED-Arc Process on the Fabrication of Invar 36 by In Situ Alloying

**DOI:** 10.3390/ma18225146

**Published:** 2025-11-12

**Authors:** Amaia Iturrioz, Juan Carlos Pereira, Eneko Ukar

**Affiliations:** 1 LORTEK Technological Centre, Basque Research and Technology Alliance (BRTA), Arranomendia Kalea 4A, 20240 Ordizia, Spain; jcpereira@lortek.es; 2 Department of Mechanical Engineering, University of the Basque Country (UPV/EHU), Ingeniero Torres Quevedo Plaza, 1, 48013 Bilbao, Spain; eneko.ukar@ehu.eus

**Keywords:** CMT-Twin DED-Arc, Invar 36, in situ alloying, critical raw material, mechanical properties, microstructure

## Abstract

This research explored the technical feasibility of creating a controlled chemical composition for Fe-Ni alloys using a Directed Energy Deposition (DED) arc metal additive manufacturing (AM) process in its twin wire feed mode. This method employs two independently controlled arc power sources to feed two different wires into a single torch, creating a unified melt pool protected by a single shielding gas nozzle. The research focused on producing Invar 36 (EN 1.3912), a low thermal expansion alloy, by melting and mixing steel and Ni-Fe wires using Cold Metal Transfer-Twin (CMT-Twin) technology. This method enables the fabrication of multi-material components featuring regions with distinct chemical compositions, including functional gradients, with the aim of leveraging the advantageous properties of each individual material. Furthermore, this new manufacturing route offers the possibility to avoid using some alloying elements, such as Nb, an element considered a critical raw material (CRM) in the European Union (EU). Microstructure and mechanical properties were analyzed and compared to commercial Invar 36 obtained by DED-Arc with single wire as well as the effect of the absence of Nb. Results showed that the in situ obtained alloy had 10–20% lower strength but exhibited 10–15% higher elongation compared to the commercial alloy, making it a promising alternative for advanced manufacturing by using this new manufacturing route.

## 1. Introduction

Metal additive manufacturing (AM) technology has attracted wide attention over the past years. This manufacturing process, which adds metallic materials to the parts layer by layer, has advantages such as design flexibility, creation of new functionalities in the parts, and short lead times. Within metal AM processes, there are the so-called Directed Energy Deposition (DED) technologies, in which a focused energy source, such as a laser beam, an electron beam, or an electric arc, are used to melt materials as they are being deposited. During the DED process, metallic powder or wires can be used.

In recent years, metal additive manufacturing (MAM) has undergone significant advances, positioning itself as a transformative technology across aerospace, automotive, and biomedical sectors. Cutting-edge research has demonstrated the superiority of MAM techniques—particularly Directed Energy Deposition (DED)—in producing complex geometries, optimizing material usage, and enabling in situ alloying strategies that are unattainable through conventional manufacturing methods [1,2].

For example, Xu et al. [3] emphasize the role of physics-based modeling in enhancing process predictability and control, which is crucial for achieving high-performance components with tailored microstructures and properties. Additionally, recent developments in multi-material AM have enabled the fabrication of functionally graded structures with customized mechanical and thermal behavior, further expanding the design freedom and application potential of metal AM technologies [4,5]. These innovations underscore the growing maturity and industrial relevance of metal AM, reinforcing its role as a superior alternative to traditional subtractive manufacturing approaches.

Nowadays, the value of novel materials in terms of economic and environmental impact is very relevant. The use of functionally graded materials (FGMs) is attracting more interest, because of their ability to have extremely functional zones in a single part, which results in novel and customized mechanical or thermal properties [6]. The advantages of enhancing interface bonding between different materials by FGMs can be easily applied to AM components. By adjusting the feedstock ratio, a gradient in microstructure and mechanical properties can be achieved [7]. AM enables the development of functionally graded structures (FGS), where material composition or mechanical properties change layer-by-layer or within specific areas of a component. Additionally, DED-Arc’s in situ material hybridization provides industries with significant benefits where weight reduction, durability, and performance optimization of key components are essential. This method allows for cost-effective production by minimizing material waste, reducing by-to-fly ratios, and streamlining advanced manufacturing processes, thus fostering innovation in component design and fabrication [8].

Using multiple wires simultaneously fed into the molten pool through a multi-wire system integrated with DED-Arc enables the seamless incorporation of various metallic materials with different composition grades or variants within a single component, which facilitates the production of FGMs with an in situ alloying strategy [9]. Conventional DED-Arc processes face limitations in controlling microstructural gradients, especially in systems like Ni–Fe, where precise thermal management is critical to avoid segregation and intermetallic formation [10]. In contrast, the CMT-Twin mode introduces a mechanically controlled droplet detachment mechanism with dual-wire feeding, which significantly reduces heat input and improves deposition stability. This enables finer control over thermal cycles, which is essential for achieving smooth compositional transitions in FGMs [11]. These capabilities make CMT-Twin a technologically critical alternative to conventional DED-Arc, especially for applications requiring precise gradient control in Ni–Fe systems and other dissimilar metal combinations [12].

The Twin Gas Metal Arc Welding (GMAW) process is characterized by the combination of two independent welding sources that are fully synchronized [13]. Two filler metals are fed into a single torch hose pack via separate feeders and routed through electrically isolated contact tips. A single shielding gas nozzle is employed, and the two arcs generate a single melt pool. The ability to isolate each power source and synchronize them allows for the implementation of Cold Metal Transfer (CMT), pulsed, or other innovative welding curves independently, leveraging a range of process combinations [14].

Invar 36 (Fe-Ni36 alloy/ASTM F1684/UNS K93600/EN 1.3912 [15]) is commonly used in molds for the fabrication of aeronautical, aerospace, and astronautics composite components and structures due to its low or nearly zero expansion coefficient (1.2 ppm/°C, compared to ordinary steels which have values around 11–15 ppm/°C). This material property is advantageous for achieving tight tolerances in molded components [16]. Currently, machining is the most prevalent manufacturing process for molds in Invar 36 alloy. However, this process faces challenges due to the alloy’s high ductility (0.06–0.45), low conductivity (12–15 W/m K), and work hardening capability, which leads to significant tool wear during machining. Invar is an attractive material for molds due to its properties, although its cost is relatively high. Owing to its distinctive properties, Invar 36 has emerged as a material of considerable interest for fabrication via DED-Arc processes, as can be seen in several recent publications [17,18,19,20,21,22]. Through AM using the tandem GMAW arc welding process, Invar can be applied in the desired areas while steel can be used for structural regions. The use of the tandem GMAW arc welding process offersthe advantages that, by using unique welding equipment and controlling the wires’ feed speed, the desired in situ alloy can be achieved. Yang et al. [23] studied the combination of twin wire arc AM with in situ alloying. They successfully created the biomedical Ti-6Al-7Nb alloy, achieving a good combination of strength and ductility. Wang et al. [24] studied the wire arc AM (DED-Arc) technique to deposit a protective NiTi layer on a Ti6Al4V substrate through in situ alloying of separate pure Ni and Ti wires to enhance the surface mechanical properties. Both studies employed GTAW (arc welding technology), which are characterized by a lower material deposition rate compared to the CMT-Twin process, and may pose limitations for large-scale industrial implementation. P. Henckell et al. [25] studied the in situ alloying using a GMAW combined with additional hot-wire feeding. In this case, two separate wires made of titanium and aluminum were used to create alloy compositions between 10 at% and 55 at% aluminum by changing the feeding rates. The results show insufficiently mixed areas of Ti and Al in samples with aluminum contents above 40 at%. This is attributed to differences in density and melting temperature between the materials, which lead to segregation within the weld bead. Klein et al. [26] investigated the possibility of using dual-wire techniques based on CMT to create alloys in the welding process in situ. A modified CMT-Twin welding system was used with standard composition grades and commercial wires differing significantly in their alloying content (AlSi7Mg and AlSi12). The study indicated that dual-wire processes were highly effective in increasing achievable deposition rates and facilitating in situ alloying. Although good overall chemical homogeneity was achieved, the silicon content did not reach the target value.

The continuous advancement of material hybridization through additive manufacturing is set to transform manufacturing capabilities in aerospace, automotive, and other industries. Future research will focus on refining deposition techniques, optimizing material combinations, and enhancing the understanding of material interfaces to fully exploit the potential of hybrid structures for superior performance and efficiency in component manufacturing.

The method proposed in this work also enables the production of alloys by eliminating undesired elements. In this case, the absence of Nb from the Invar 36 alloy is studied, as Nb is considered a critical raw material (CRM) by the European Union (EU). Niobium is classified as a CRM due to its high supply risk—over 90% of global reserves are concentrated in Brazil, and its extraction is dominated by a single company, which increases vulnerability to geopolitical and market disruptions [27]. Moreover, the recycling rate of niobium remains low, and its substitutability is moderate, making its sustainable management a priority for the EU. Eliminating Nb from alloy design contributes to reducing CRM dependency, and aligns with the EU’s Critical Raw Materials Act, which promotes diversification, substitution, and circularity in strategic sectors. The EU aims at promoting a circular economy by encouraging the sustainable design of products, increasing the use of secondary raw materials, and enhancing recycling and reuse processes. From an environmental perspective, niobium extraction and processing are associated with high energy consumption and significant environmental impacts, including abiotic resource depletion and freshwater ecotoxicity, especially when using conventional methods involving hydrofluoric acid [28]. Therefore, alloy designs that avoid Nb not only support strategic autonomy but also contribute to lower life-cycle environmental burdens, reinforcing the EU’s goals for a cleaner and more resilient industrial ecosystem. This approach seeks to reduce dependency on primary CRM and minimize environmental impact. In parallel, the EU is committed to advancing research and innovation in the field of raw materials, focusing on waste management, advanced materials, and replacement strategies [29].

This study analyses the feasibility of creating Invar 36 alloy through the in situ alloying of two wires using the tandem GMAW arc welding process and CMT welding curve.

## 2. Materials and Methods

**Figure 1:**
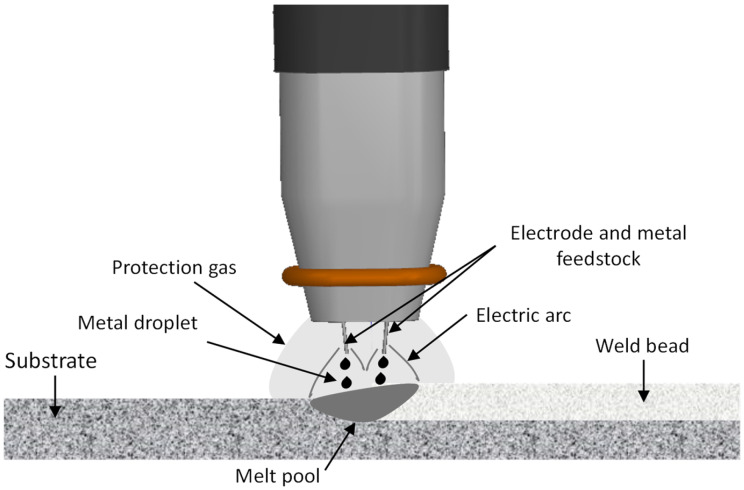
Scheme of CMT-Twin two electric arcs.

Invar 36 in situ alloyed parts (single walls of 230 × 130 mm) were produced using an arc welding robotic cell. The equipment included Fronius TransPlus synergic (TPS) 4000 CMT R and 5000 CMT R power sources, both fully digital inverter CMT welding power sources, and a Robacta Twin Compact Pro 30° PA OVT torch from Fronius International (Wels, Austria). Figure 1 shows the torch and the generated electric arc.

**Figure 2:**
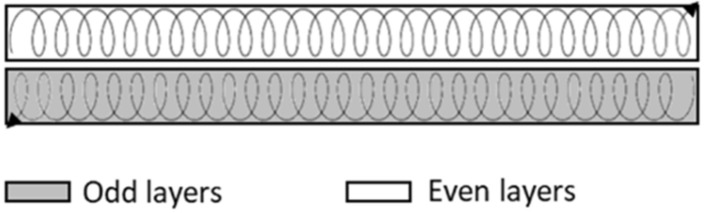
Details for the building strategy followed for DED-Arc wall.

The welding torch was attached to a Fanuc ARC Mate 120iC robot (Fanuc Corporation, Oshinomura, Yamanashi Prefecture, Japan). The gas shielding of the torch was performed with a mixture of Ar (98%) and CO_2_ (2%) and the gas flow was set at 30 L/min. The orientation of the torch was perpendicular to the substrate. The arc length was set to 15 mm and the offset in Z direction was continuously adjusted in a range of 2–2.5 mm in order to maintain a constant voltage. Travel speed (TS) was fixed at 8 mm/s during the whole deposition process. In this case, as can be seen in Figure 2, the strategy used was the circular single weld bead [30], in which the material deposition is carried out using a single weld bead per layer but with a circular movement in its trajectory, in which the torch oscillates to generate an overlap of round circles with 2 mm amplitude and 3 Hz frequency. The dwell time between the deposition of consecutive layers was 110 s. These parameters were previously optimized through experimental trials.

As filler metal, two commercial wires were used, a FN55 alloy manufactured by VBC group and a ER70-S6 wire manufactured by WSD, both with a diameter of 1.2 mm. The chemical composition provided by the manufacturer is shown in Table 1. Substrates of S235 steel, obtained by hot rolling, with a thickness of 10 mm, were used for the manufacturing of the walls. These substrates were clamped to the positioning table.

To obtain the desired chemical composition, FN55 and ER70-S6 wires were simultaneously fed into the melt pool, with the proportion of their constituents regulated by the respective wire feed speeds (WFS) based on mass flow control (g/min). The mass fraction ($E_{X}$) of the main alloying elements in the deposited material can be calculated using the following equation, Equation (1) [9]:

(1) $$E_{X}=\frac{\sum {WFS}_{i}d_{i}\rho_{i}E_{xi}}{\sum {WFS}_{i}d_{i}\rho_{i}}$$

where $E_{xi}$ (x = Fe, Ni; i = 1, 2) is the mass fraction of a certain element in a distinct wire, ${WFS}_{i}$ (i = 1, 2) is the wire feed rate in mm/min, $d_{i}$ (i = 1, 2) is the wire diameter in mm, and $\rho_{i}$ (i = 1, 2) is the density of the wire in g/cm^3^.

Applying the described formula, a WFS ratio of 1.7:1 for FN55 to ER70-S6 was established. Using this ratio, various tests were conducted while consistently maintaining this WFS proportion. Table 2 summarizes the WFS ratio parameters that provided the best outcomes regarding geometry and process stability. These parameters were previously optimized through experimental trials considering the adequate melt and deposition of the materials, wall growth stability, surface finish, dimensional stability, and high deposition rates. These parameters were employed in the fabrication of walls A and B. The theoretical heat input is calculated following Equation (2):

(2) $$HI=\frac{I_{1}\times V_{1}+I_{2}\times V_{2}}{TS}$$

where I is the instantaneous current (A), V is the instantaneous voltage (V), and TS is the travel speed (mm/s); 1 refers to the FN55 wire and 2 refers to the ER70-S6 wire.

Table 3 summarizes the predicted chemical composition in wt.% of the two samples for Invar 36 in situ obtained alloy (Sample A and Sample B). As observed, the theoretical Ni content is below 36 wt.%, being 35.346 wt.% for Sample A and 34.076 wt.% for Sample B. This discrepancy arises from preliminary tests, which consistently show that the actual Ni percentage obtained is higher than the theoretical value, with previous studies measuring Ni content in fabricated samples via ICP indicating that the real value was between 6 and 7% higher than the theoretical one. One plausible explanation is the preferential incorporation of Ni during solidification, as described in the work by Chiareli et al. [31], where the segregation coefficient and density-driven flow during alloy solidification can lead to macro segregation and enrichment of Ni in the solid phase. Additionally, oxidation behavior under DED-Arc conditions may contribute to this imbalance. Iron is more prone to oxidation than nickel in high-temperature environments, especially in atmospheres with residual oxygen, leading to selective loss of Fe during deposition. This phenomenon has been extensively studied in the context of additive manufacturing, where Ni-based alloys show superior oxidation resistance due to the formation of stable oxide layers, while Fe tends to form less protective oxides that can volatilize or be lost from the melt [32,33].

**Figure 3:**
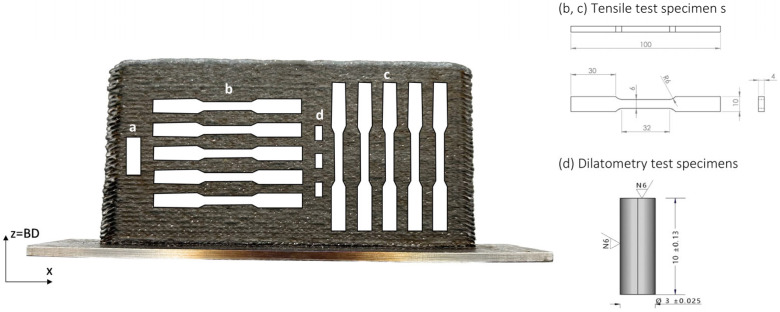
Scheme of the specimens (front view) for the characterization to be performed: (a) specimen for microstructural, compositional, and hardness analysis; (b) tensile test specimens from the horizontal orientation; (c) tensile test specimens from the vertical orientation; and (d) dilatometry test specimens.

Once the Invar 36 in situ alloyed wall was manufactured, samples for characterization were cut (see Figure 3). A 5 mm thick section was taken at mid-height in the building direction from the manufactured wall (a) for the microstructural, compositional, and hardness analysis. Ten tensile test specimens were obtained using Electric Discharge Machining (EDM) method, five from the horizontal orientation (b) and five from the vertical orientation (c), according to the ASTM E8M standard. To perform dilatometry tests, three specimens were machined in the growth direction of the wall (d).

Specimens for microstructural, compositional, and hardness analysis were cut, mounted, ground, polished, and etched with Kalling N° 2 (0.5 g de CuCl_2_, 100 mL de HCl, 100 mL de Ethanol). Once the samples were prepared, advanced microstructural and compositional characterization was performed by light microscopy using an Olympus GX51, Olympus Corporation, Tokyo, Japan and by field emission gun scanning electron microscopy (FEG-SEM) using a ZEISS Ultra Plus Field Emission, ZEISS, Overkochen, Germany.

**Figure 4:**
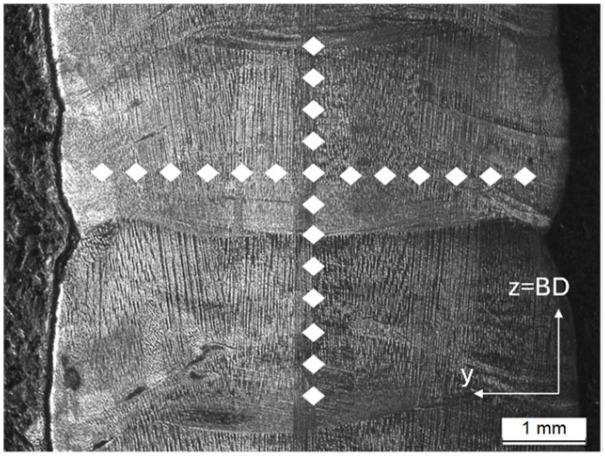
Scheme of the position of the hardness measurements (side view) performed in the wall.

Hardness test was carried out using an EmcoTest Durascan 20 micro-durometer, Emco-Test Prüfmaschinen GmbH, Kuchl, Austria. The measurements were taken as shown in Figure 4.

Tensile tests were performed using a Z100 ZWICK/Roell model testing machine, ZWICK/Roell, Ulm, Germany, with a maximum load capacity of 100 kN. Specimens were tested at room temperature with a displacement rate of 1.6 mm/min and using an extensometer with a 30 mm gauge length.

Dilatometry analyses were conducted using a Bähr DIL805A/D, TA Instruments Inc., New Castle, United States, deformation and cooling dilatometer, equipped with the Alpha measurement system (resolution ΔL/°C = 0.05 mm/0.05 °C). The Alpha measuring head incorporates low-expansion fused silica components in conjunction with a true differential Linear Variable Differential Transformer (LVDT), enabling high-precision thermal expansion measurements. This configuration facilitates accurate determination of the coefficient of thermal expansion (CTE). Specimens consisting of solid cylindrical samples with a diameter of 4 mm and a length of 10 mm were analyzed. The thermal cycle involved rapid heating of the specimens to a target temperature of 1000 °C, at a controlled heating rate of 5 °C/s. Temperature was measured via a type S thermocouple, and all experiments were carried out under an inert argon atmosphere to prevent oxidation and ensure thermal stability.

## 3. Results

### 3.1. Morphological Characterization

**Figure 5:**
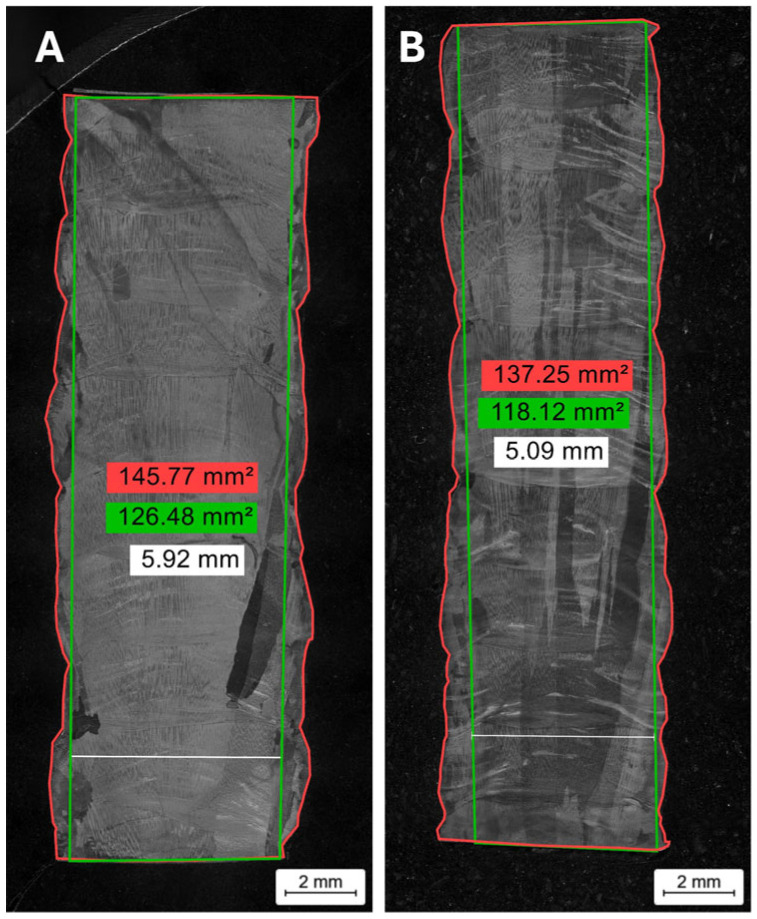
Morphological characterization of Samples A and B. The total area is indicated in red and the effective area without waving is indicated in green. These values were used to calculate the buy-to-fly ratio. The effective width is indicated in white.

The external geometry of the manufactured walls has been analyzed. Figure 5 shows the total area (red), obtained directly from the manufacturing process, and the effective area without corrugation (green). The area that can be achieved by machining the external undulations and waviness (the largest rectangle that can be formed in the cross-section) has also been indicated. These area values are used for the buy-to-fly ratio (BTF) calculation (Table 4) for Samples A and B. The buy-to-fly ratio refers to the relationship between the starting material and the finished part and is calculated by dividing the weight of the raw material by the weight of the final component. The effective width of both samples is indicated in white. Table 4 also shows the values from a previous study [30] on the manufacturing of Invar 36 using DED-Arc with a commercial wire, with the aim of comparing the morphological results between using a single wire and a mixture of two wires.

### 3.2. Microstructural Characterization

**Figure 6:**
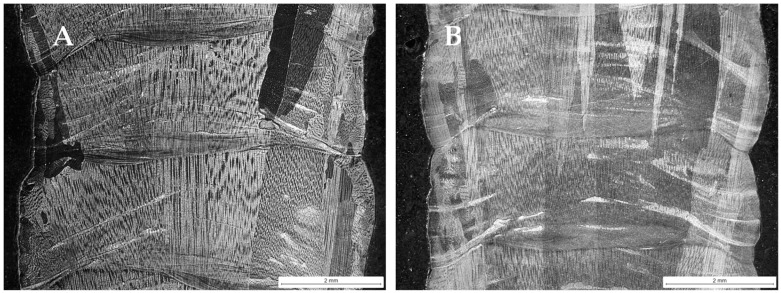
Cross-section of Invar 36 in situ alloyed DED-Arc walls after cut for testing and analysis (Sample (**A**) on the left, Sample (**B**) on the right).

A cross-section of the manufactured walls in the Invar 36 in situ alloy is shown in Figure 6, where the fusion lines of the deposited layers can be observed. Elongated, large grains that grow in the building direction can be seen. These columnar grains extend through multiple layers.

**Figure 7:**
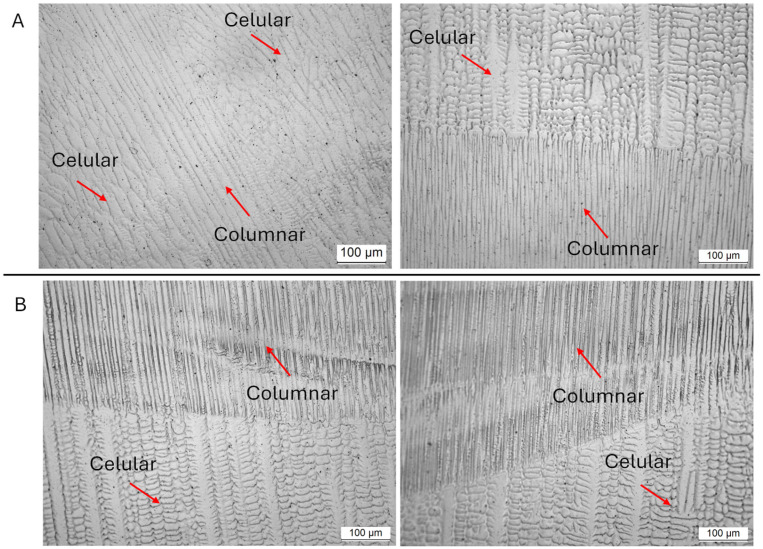
LOM micrographs for substructure analysis of the Invar 36 in situ alloyed DED-Arc wall (Sample (**A**) on top, Sample (**B**) down).

Figure 7 shows the complementary microstructural analysis, carried out by Light Optical Microscopy (LOM), where the cellular and columnar dendrites of the substructure can be seen.

**Figure 8:**
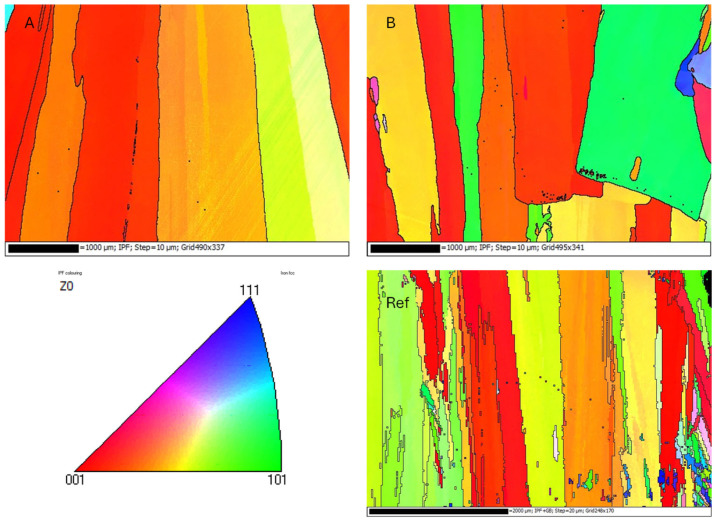
EBSD inverse pole figure (IPF) maps of Sample (**A**) (top left), Sample (**B**) (top right), and conventional (below) Invar 36 alloy manufactured by DED-Arc using a circular single weld bead strategy [29].

Figure 8 shows the Electron Backscatter Diffraction (EBSD) inverse pole figure (IPF) maps of the in situ alloyed DED-Arc walls (A and B) and a map of a conventional Invar 36 alloy manufactured by DED-Arc using a circular single weld bead strategy [30], which is the strategy used for manufacturing the in situ alloyed walls. The three cases are characterized by a columnar grained structure aligned parallel to the build direction. In the in situ alloyed cases, the grain width is more than 1 mm in the largest grains, which is twice that observed in conventional Invar 36. Notably, the in situ Invar 36 alloy is Nb-free. In the three cases, the crystallographic orientation ranges from (001) to (101).

**Figure 9:**
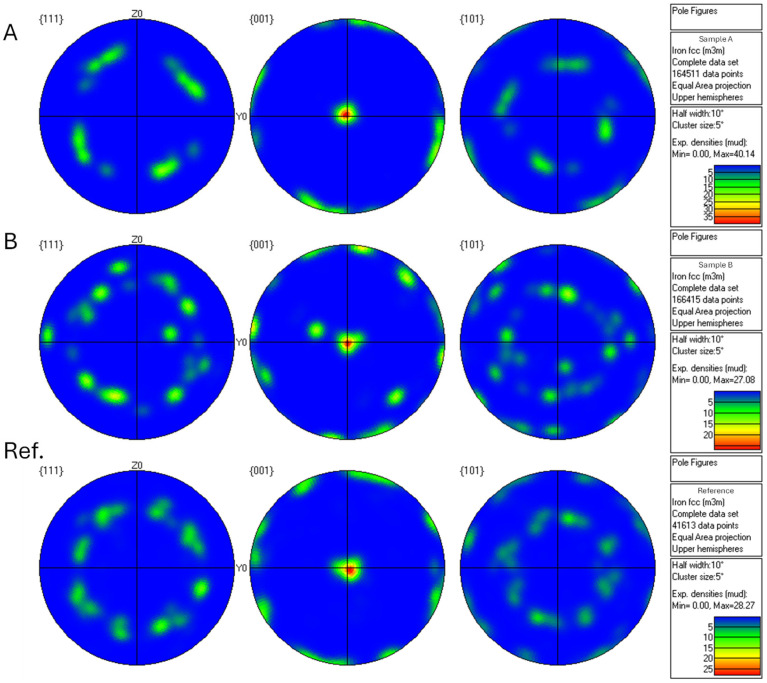
Pole figures of Sample A, Sample B, and conventional Ref. Invar 36 alloy manufactured by DED-Arc using a circular single weld bead strategy.

Additionally, pole figures corresponding to the {111}, {001}, and {101} planes are shown in Figure 9 to further clarify the crystallographic texture evolution in the three samples. These figures reveal that all cases exhibit a preferred orientation along the build direction, consistent with the columnar grain structure observed in the IPF maps. Samples A and B, which were fabricated with higher thermal input, show stronger texture intensity compared to the conventional Invar 36 alloy. Despite the similar chemical composition between Samples Ref and B, their pole figures display comparable orientation distributions. In contrast, Sample A presents a more pronounced texture, likely due to the increased thermal input during deposition. These results confirm that both composition and thermal energy input play a significant role in the development of the crystallographic texture in Fe-Ni alloys processed via DED-Arc.

### 3.3. Chemical Characterization

Inductively Coupled Plasma (ICP) elemental measurements were performed in the manufactured Invar 36 in situ walls (see results in Table 5). The Ni percentage measured in case A is 38.5 wt. % whereas in case B, 36.3 wt. % was obtained. As previously mentioned, a discrepancy between the theoretical and actual results has been observed. This difference must be considered during the manufacturing process to ensure accurate control of the final composition.

**Figure 10:**
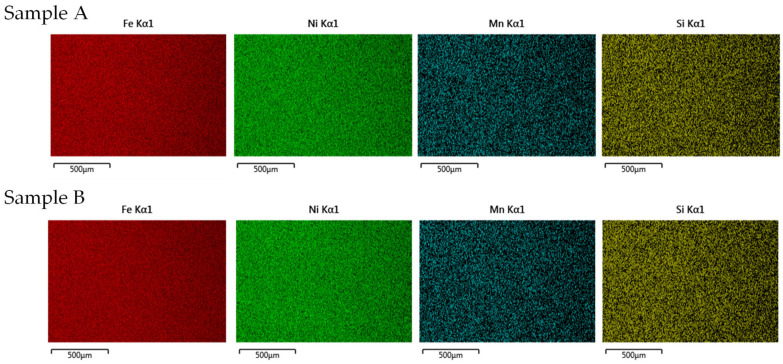
EDS elemental measurement where Fe, Ni, Mn, and Si distribution is shown; Sample A (**top**) and Sample B (**bottom**).

Figure 10 shows the EDS (Energy dispersive spectrometry) elemental measurement for Samples A (top) and B (bottom). The mapping images indicate that wire mixing through CMT-Twin DED-Arc leads to a homogeneous elemental distribution. As expected for Invar 36, which is a single-phase solid solution, no precipitate phases are observed.

### 3.4. Mechanical Characterization

Table 6 shows the summary of mechanical properties obtained for the Invar 36 in situ alloyed walls. Mechanical properties were obtained for the Z (vertical direction) and X (horizontal direction) directions. Average values for all samples and standard deviations are given. The results are compared to those obtained in a previous study using the same manufacturing strategy (circular single weld bead [30]) with conventional Invar 36 and with references from the literature. Anisotropy was evident, with the horizontal direction (X) consistently exhibiting greater strength and reduced elongation. The properties exhibited by the in situ alloys (A and B) are inferior to those reported in previous studies [30,34,35] employing conventional Invar 36.

### 3.5. Hardness

Hardness measurement tests were carried out on the cross-sections on the Z (vertical or build direction) and X (horizontal or transverse direction) directions of the Invar 36 in situ alloyed DED-Arc parts (see Table 7).

### 3.6. Dilatometry Analysis

Dilatometry tests were conducted along the Z-axis (vertical direction) to characterize the thermal behavior of the A and B samples. For each wall produced, three specimens were sectioned in the Z direction to compare the behavior of in situ alloyed Invar 36 with that of conventionally manufactured Invar 36 DED-Arc components. During the dilatometry tests, specimens were subjected to controlled heating and cooling cycles, while their linear displacement was continuously measured. The CTE, expressed in units of 10^−6^ °C^−1^, was calculated using (Equation (3)).

(3) $$CTE=\alpha =\frac{∆L}{L_{0}\times ∆T}$$

where ∆L is the linear deflection, $L_{0}$ the initial length of the specimen, and ∆T the temperature difference.

**Figure 11:**
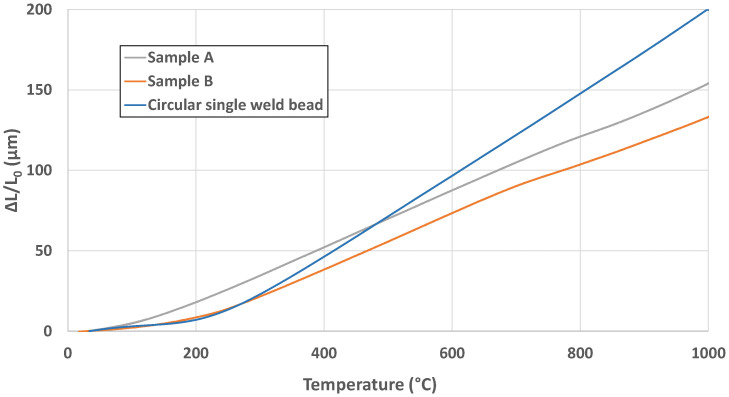
Dilatometry curves of Samples A and B compared to the circular single weld bead strategy with conventional Invar 36.

Figure 11 presents the average heating curves obtained from the dilatometry tests conducted on Samples A and B, and from the reference material (circular single weld bead, [30]), which was also fabricated using the DED-Arc process and the same manufacturing strategy as Samples A and B. The dilatometry curves values obtained do not match those reported in the previous study. Among the current specimens, Sample B shows the closest agreement, particularly at lower temperatures. This behavior is likely related to its chemical composition, which most closely resembles that of standard Invar 36.

**Figure 12:**
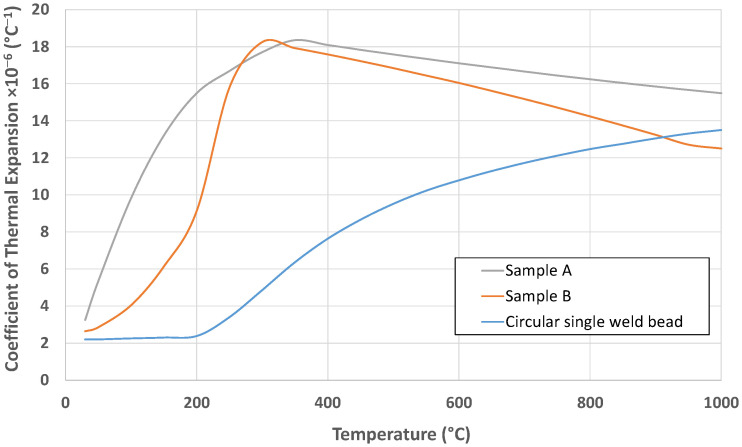
CTE value evolution in relation to temperature.

Figure 12 shows the CTE value evolution with respect to temperature. The results obtained for Samples A and B were compared with data from a previous study [30]. As in the case of the dilatometry curves, the CTE values differ from the reference material. Sample B is closer to the reference at the lower temperatures.

## 4. Discussion

In this study, the feasibility of manufacturing an Invar 36 in situ alloy avoiding the addition of Nb by mixing steel and Ni-Fe wires using CMT-Twin technology has been investigated.

The morphological characterization clearly proves that, with an optimal control of the parameters, the same level of detail can be achieved using a single wire or a Twin mode, using in both cases the circular single weld bead strategy [30]. It should be noted that in none of the cases were defects such as cracks or lack of fusion observed.

The microstructural characterization reveals that under the same manufacturing strategy (circular single weld bead), both single and double arc wire processes result in a columnar grain structure aligned parallel to the build direction (Z-axis). However, in Twin mode, larger grain widths are obtained. This may be due to several reasons. One factor that may contribute to the observed differences is the effect of heat input. The Twin welding mode employs two electric arcs, which can significantly increase the total energy delivered to the material. In the present study, the heat input values for Samples A and B were 316.24 KJ/cm and 316.37 KJ/cm, respectively. These values are notably higher than those reported in a previous study using the same deposition strategy but with a single arc, where the heat input was 204 KJ/cm [30]. Higher heat input typically leads to a slower cooling rate, which in turn affects the solidification dynamics. Specifically, a lower temperature gradient (G) and a reduced solidification rate (R) result in a lower G/R ratio. According to solidification theory, a lower G/R ratio promotes the growth of coarser columnar grains. This phenomenon explains the larger grain widths observed in the Twin mode. Köhler et al. [36] demonstrated that increased energy input in DED-Arc processes leads to pronounced grain growth and softening zones, particularly along the build direction. Similarly, Reindl et al. [37] emphasized that thermal gradients and heat accumulation significantly influence microstructural uniformity and hardness in DED-Arc components. Nor Ana Rosli et al. [38] conducted a comprehensive review on the influence of heat input across eight different arc welding technologies. Their findings underscore that variations in heat input significantly affect the microstructural characteristics, such as grain size, of the components manufactured by DED-Arc. Another factor that affects the grain size is the absence of the Nb alloying element. Niobium acts as a grain-refining element by forming carbides or intermetallic compounds that hinder grain growth during solidification [39]. Cuixin Chen et al. [21] examined the influence of Cr, Mo, V, and N in the grain refinement, beyond its influence on other properties. The study concluded that these elements precipitate in the high-strength Invar 36 alloy manufactured by DED-Arc, refining the grain and cell substructure and contributing to the strength of the material. This could represent a viable alternative to the non-use of Nb in this alloying system.

Regarding chemical composition, the EDS results indicate that this method is suitable for producing alloys from mixed wires. The analysis confirms that the wires are homogeneously mixed during the process, without the formation of regions with distinct chemical compositions. This uniformity is essential for ensuring consistent material properties throughout the deposited structure. Even though a chemical composition similar to Invar 36 alloy has been achieved (Invar 36 in situ alloy B), differences between theoretical and obtained real chemical compositions indicate that more process control is needed.

The mechanical properties of the created in situ alloys are inferior compared to previous studies using the same manufacturing strategy and conventional Invar 36 alloy [30] and also to studies in the literature [34,35]. When comparing the results of the B sample, whose chemical composition coincides more closely with that of Invar 36, it can be seen that its properties are still lower than those obtained for the same conventional alloy. This discrepancy may be attributed to the larger grain size and the absence of Nb. Comparative analysis with mechanical properties obtained confirmed that both Sample A and Sample B exhibited lower yield strength (Rp0.2) and ultimate tensile strength (Rm) compared to the reference alloy in both Z and X orientations. Sample A also showed higher elongation in the Z orientation, while Sample B did not show differences in elongation. When comparing Samples A and B, differences in strength were evident, particularly in Rm and elongation in the Z orientation, suggesting that even small compositional or microstructural variations can lead to measurable mechanical differences. Pole figure analysis supports this interpretation, revealing that Samples A and B—fabricated with higher thermal input—exhibit stronger crystallographic texture and larger columnar grains aligned with the build direction. Sample A, in particular, shows the highest texture intensity and the largest grain width, which correlates with its increased elongation and reduced strength.

Van-Thuc Nguyen et al. [40] analyzed the effect of the process parameters in the mechanical properties of the DED-Arc manufactured parts. They concluded that with smaller grain size, higher values of ultimate tensile strength (UTS) are observed. The opposite occurs in this work: with larger grain size, both the mechanical properties and the hardness of the material decrease. Moreover, as is well known, the addition of niobium as an alloying element improves the mechanical properties [41,42]. This Nb is usually present as precipitated particles that act as barriers to dislocation movement, thereby increasing the strength. As the in situ alloy achieved in this study does not have Nb, there are no precipitated particles that act as dislocation barriers.

Regarding the dilatometry curves and CTE values measured for the in situ alloys, it is observed that Sample B is the one that has most closely matched the result obtained in the previous study with conventional Invar 36, although this behavior remains far from that of Invar 36. At 100 °C, the dimensional change (ΔL/L_0_) for Sample A is approximately 15 µm, compared to 10 µm for the reference material and 8 µm for Sample B. At 200 °C, Sample A reaches about 35 µm, while both the reference material and Sample B show similar values around 20 µm. In terms of CTE, at 100 °C Sample A exhibits ~8 × 10^−6^ °C^−1^, significantly higher than the reference (~3 × 10^−6^ °C^−1^) and Sample B (~4 × 10^−6^ °C^−1^). At 200 °C, Sample A peaks at ~14 × 10^−6^ °C^−1^, while Sample B reaches ~10 × 10^−6^ °C^−1^, both exceeding the reference value of ~3 × 10^−6^ °C^−1^. These results indicate that Sample A shows the greatest thermal expansion and CTE increase at low temperatures, whereas Sample B remains closer to the reference material in terms of dimensional stability. Aldalur et al. [43] compares the results of manufacturing Invar 36 alloy by DED-Arc process using PAW (Plasma arc welding) and GMAW processes, in which the CTE results of the PAW process are higher due to the higher heat input. Considering that in this study the fabrications were carried out with a higher heat input, this could be the reason for the higher CTE values observed. Genghao Jiao et al. [44] observed differences in CTE values between samples with Ni contents of 36.4 wt. % and 35.72 wt. %, obtaining better CTE values with the second Ni content. This sensitivity is linked to the magneto volumetric behavior of Fe–Ni systems, where spontaneous volume magnetostriction below the Curie temperature compensates for lattice shrinkage due to cooling. A Ni content closer to 36 wt. % enhances this compensation effect, resulting in lower CTE values. The CTE of the Invar 36 alloy is very sensitive to the Ni content, so better control of the resultant chemical composition is needed.

**Figure 13:**
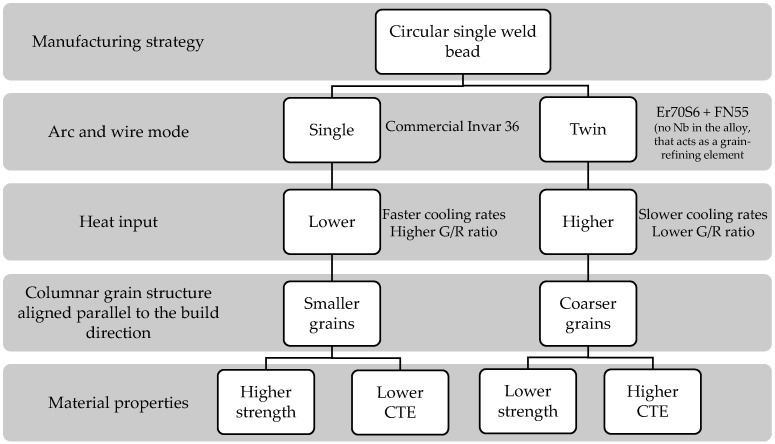
Influence of arc mode and heat input on microstructure and properties of in situ Invar 36 alloy obtained.

In summary, the results demonstrate that the arc mode and associated heat input play a critical role in determining the microstructural and mechanical behavior of the in situ Invar 36 alloy. Higher heat input in Twin mode leads to slower cooling rates and a lower G/R ratio, promoting coarser columnar grains aligned with the build direction. These microstructural features, combined with the absence of Nb, explain the reduced strength and increased CTE observed compared to Single mode, which produces finer grains under lower heat input and faster cooling rates. Figure 13 schematically summarizes these relationships, highlighting the link between manufacturing parameters, solidification dynamics, and material properties.

## 5. Conclusions

In this study, Invar 36 in situ alloy DED-Arc manufactured parts were obtained, having some differences with the commercial Invar 36 DED-Arc manufactured parts in the analyzed characteristics, composition ranges, and properties:

- The same level of effective area and BTF ratio can be obtained by DED-Arc with commercial Invar 36 alloy and Twin DED-Arc with wire mixing in Sample B, obtaining an effective area of 81.2% and a BTF ratio of 1.23 in the first case, and an effective area of 86.1% and a BTF ratio of 1.16 in the second case.
- A very close chemical composition to the standard Invar 36 alloy (Fe-Ni36 alloy/ASTM F1684/UNS K93600/EN 1.3912) has been achieved in Sample B in this study, with a Ni content of 36.3 wt.%. However, deviations from the theoretical composition highlight the need for tighter process control, especially considering the sensitivity of thermal expansion behavior to Ni content.
- The high heat input of the Twin feed DED-Arc process using the CMT welding curve has resulted in larger grain size (twice that observed in conventional Invar 36 by CMT DED-Arc), poorer mechanical properties (with 10–20% lower strength but 10–15% higher elongation) and higher CTE values. The focus of this study was set in the achievement of the chemical composition.
- Sample B exhibited thermal expansion and CTE values closer to those of conventional Invar 36, although still higher than desired. Sample A showed markedly higher CTE values, likely due to its larger grain size and higher heat input. These findings reinforce the importance of precise control over both composition and thermal input during manufacturing.

In summary, while the in situ alloying approach shows promise for producing Invar 36-like materials, further optimization is required. Future work should focus on refining heat input strategies—such as reducing deposition rates or incorporating external cooling—and enhancing compositional control to improve mechanical and thermal performance.

## Figures and Tables

**Table 1:** Chemical composition (wt.%) of Ni55Fe (provided by VCB Group) and ER70-S6 (provided by WSD) wires feedstock.

Materials	Si	Mn	P	Cr	Ni	S	Cu	Al	V	Co	Mo	C	Fe
FN55	0.11	0.66	0.003	0.013	54.96	0.002	0.012	0.007	---	0.073	---	0.015	44
ER70-S6	0.86	1.447	0.018	0.038	0.04	0.018	0.069	0.002	0.003	---	0.012	0.064	Bal

**Table 2:** Welding parameters for DED-Arc Invar 36 in situ alloying parts manufacturing.

Sample	Wire	Wire Feed Speed (m/min)	Theoretical Mass Flow (g/min)		Current (A)	Voltage (V)	Heat Input (KJ/cm)
Sample A	FN55	5.2	49.3	76.7	161	11.8	316.24
	ER70-S6	3.1	27.3		120	10.6	
Sample B	FN55	5.0	47.3	76.45	157	11.7	316.37
	ER70-S6	3.3	29.2		124	10.7	

**Table 3:** Predicted chemical composition in wt.% of the Invar 36 in situ obtained alloy.

Sample	Si	Mn	P	Cr	Ni	Cu	Co	Fe
Sample A	0.378	0.941	0.008	0.008	35.346	0.008	0.047	Bal
Sample B	0.126	0.625	0.008	0.008	34.076	0.006	0.006	Bal

**Table 4:** Calculated effective area, BTF ratio, and effective width.

Strategies Used	Calculated Effective Area (%)	BTF Ratio	Effective Width
	(Effective Area/Total Area) ×100	Total Area/Effective Area	Without Waving
Sample A	86.7	1.10	5.9
Sample B	86.1	1.16	5.1
Circular single weld bead [30]	81.2	1.23	5.8

**Table 5:** ICP chemical composition in wt.% of the Invar 36 in situ obtained alloy.

Materials	Si	Mn	P	Ni	Fe
Predicted Sample A	0.378	0.941	0.008	35.346	Bal
ICP Sample A	0.320	0.870	0.160	38.500	Bal
Predicted Sample B	0.126	0.625	0.008	34.076	Bal
ICP Sample B	0.320	0.960	0.010	36.300	Bal

**Table 6:** Mechanical properties (Rp0.2: yield strength, Rm: ultimate tensile strength, e: elongation) achieved for both orientations for DED-Arc parts manufactured using different strategies.

Strategies Used	Orientation	Rp0.2 (MPa)	Rm (MPa)	e (%)
Sample A	Z	218.6 ± 2	363.3 ± 2.5	36.3 ± 1.7
	X	267.5 ± 7	438.1 ± 3	27.5 ± 4
Sample B	Z	226.45 ± 4	370.4 ± 1	31.8 ± 2.1
	X	267.2 ± 7	415.2 ± 4	29.3 ± 2.6
Circular single weld bead [30]	Z	310 ± 3.6	460 ± 1.6	30 ± 1.6
	X	339 ± 4.2	484 ± 1.4	27 ± 1.9
Reported in Bibliography [34,35]	Z	305 ± 6	464 ± 9	33 ± 1
	X	345 ± 5	505 ± 2	29 ± 1

**Table 7:** Microhardness values of the Invar 36 in situ alloyed DED-Arc walls in the Z (vertical direction) and X (horizontal direction) directions.

Wall	Orientation	HV0.1
Sample A	Z	154.7 ± 2.83
	X	157 ± 4.71
Sample B	Z	156.90 ± 13.73
	X	154.38 ± 8.16

## Data Availability

The raw data supporting the conclusions of this article will be made available by the authors on request.

## Abbreviations

The following abbreviations are used in this manuscript:

DED	Directed Energy Deposition
AM	Additive Manufacturing
CMT	Cold Metal Transfer
CRM	Critical Raw Material
EU	European Union
FGM	Functionally Graded Materials
FGS	Functionally Gradient Structures
GTAW	Gas Tungsten Arc Welding
GMAW	Gas Metal Arc Welding
TS	Travel Speed
WFS	Wire Feed Speed
EDM	Electric Discharge Machining
LVDT	Linear Variable Differential Transformer
CTE	Coefficient of Thermal Expansion
BTF	Buy-to-Fly Ratio
LOM	Light Optical Microscopy
EBSD	Electron Backscatter Diffraction
IPF	Inverse Pole Figures
ICP	Inductively Coupled Plasma
EDS	Energy Dispersive Spectrometry
PAW	Plasma Arc Welding
